# Cored in the act: the use of models to understand core myopathies

**DOI:** 10.1242/dmm.041368

**Published:** 2019-12-19

**Authors:** Aurora Fusto, Louise A. Moyle, Penney M. Gilbert, Elena Pegoraro

**Affiliations:** 1 Department of Neuroscience, University of Padua, Padua 35128, Italy; 2 Donnelly Centre, University of Toronto, Toronto, ON M5S3E1, Canada; 3 Institute of Biomaterials and Biochemical Engineering, University of Toronto, Toronto, ON M5S3G9, Canada; 4 Department of Cell and Systems Biology, University of Toronto, Toronto, ON M5S3G5, Canada; 5 Department of Biochemistry, University of Toronto, Toronto, ON M5S1A8, Canada

**Keywords:** Core myopathy, Disease model, Skeletal muscle

## Abstract

The core myopathies are a group of congenital myopathies with variable clinical expression – ranging from early-onset skeletal-muscle weakness to later-onset disease of variable severity – that are identified by characteristic ‘core-like’ lesions in myofibers and the presence of hypothonia and slowly or rather non-progressive muscle weakness. The genetic causes are diverse; central core disease is most often caused by mutations in ryanodine receptor 1 (*RYR1*), whereas multi-minicore disease is linked to pathogenic variants of several genes, including selenoprotein N (*SELENON*), *RYR1* and titin (*TTN*). Understanding the mechanisms that drive core development and muscle weakness remains challenging due to the diversity of the excitation-contraction coupling (ECC) proteins involved and the differential effects of mutations across proteins. Because of this, the use of representative models expressing a mature ECC apparatus is crucial. Animal models have facilitated the identification of disease progression mechanisms for some mutations and have provided evidence to help explain genotype-phenotype correlations. However, many unanswered questions remain about the common and divergent pathological mechanisms that drive disease progression, and these mechanisms need to be understood in order to identify therapeutic targets. Several new transgenic animals have been described recently, expanding the spectrum of core myopathy models, including mice with patient-specific mutations. Furthermore, recent developments in 3D tissue engineering are expected to enable the study of core myopathy disease progression and the effects of potential therapeutic interventions in the context of human cells. In this Review, we summarize the current landscape of core myopathy models, and assess the hurdles and opportunities of future modeling strategies.

## Abstract (precis)

**Summary:** The core myopathies are neuromuscular disorders with no cure. In this Review, we outline our current understanding of pathomechanisms, the advantages and limitations of available models, and discuss emerging modeling technologies.

## Introduction

The core myopathies are the most prevalent subgroup of congenital myopathies (Amburgey et al., 2011; Gonorazky et al., 2018; Maggi et al., 2013; Norwood et al., 2009), comprising central core disease (CCD; Box 1) and multi-minicore disease (MmD; Box 2). Both disorders are clinically heterogeneous but present characteristic histological features upon muscle biopsy. Whilst the severity of CCD and MmD varies, the clinical symptoms are rather stable and do not progress with age (Dubowitz and Roy, 1970; Patterson et al., 1979; Quane et al., 1993), unlike with the muscular dystrophies, where muscle wasting and weakness are progressive (Emery, 2002). Nevertheless, the core myopathies are associated with a relatively high disease burden (van Ruitenbeek et al., 2019) and, currently, there are no approved drugs available.

**Box 1. Focus on central core disease (CCD)**

First described by Shy and Magee in 1956, CCD is the most common congenital myopathy (Shy and Magee, 1956). CCD is generally characterized by delayed motor development and non-progressive proximal muscle weakness that appears in infants or young children and can, in rare cases, be associated with ‘floppy babies’ (Shuaib et al., 1987; Shy and Magee, 1956). Weakness and atrophy of the lower limbs, pelvic girdle and shoulder girdle are common, with facial weakness observed in more severely affected patients (Shuaib et al., 1987). In most cases, patients achieve the ability to walk independently (Jungbluth, 2007). Penetrance may vary between affected family members, from severe muscle weakness to mild non-progressive weakness and sometimes non-specific neuromuscular complaints, such as fatigue following running, climbing stairs or other exerting activity (Dubowitz and Roy, 1970; Patterson et al., 1979; Quane et al., 1993). Selective muscle involvement has been observed by MRI (Box 3) in CCD families. Therefore, MRI could be a useful tool for differential diagnosis in congenital myopathies (Jungbluth et al., 2004). CCD is not associated with necrosis, myotonia or elevated serum creatine kinase (Jungbluth et al., 2011; Shuaib et al., 1987; Shy and Magee, 1956). Cardiac involvement is rare, although mitral valve prolapse and arrhythmia have been reported in some families (Shuaib et al., 1987). Respiratory involvement is also uncommon (reviewed in Jungbluth, 2007). CCD is associated with several orthopedic abnormalities, which do not correlate to the severity of the myopathy. These include kyphoscoliosis (Box 3), foot deformities and joint abnormalities, and occasionally joint contractures (Gamble et al., 1988; Quane et al., 1993; Shuaib et al., 1987; Shy and Magee, 1956). Clinical severity in CCD largely depends on the underlying gene mutation.

**Box 2. Focus on multi-minicore disease (MmD)**

First described by Engel and colleagues (Engel, 1967), MmD is an early-onset autosomal recessive congenital myopathy characterized by hypotonia, delayed motor development and general-to-proximal weakness that is stable or slowly progressive (Ferreiro et al., 2002a,b; Jungbluth et al., 2005). Like CCD, MmD can result in orthopedic abnormalities such as kyphoscoliosis (Ferreiro et al., 2002a,b; Jungbluth et al., 2005) but, unlike CCD, it often has respiratory involvement (Ferreiro et al., 2000; Shy and Magee, 1956). Diagnosis is confirmed by the presence of multiple mitochondria-depleted minicores within biopsied myofibers (Engel et al., 1971). MmD is not associated with necrosis or fatty or fibrotic infiltration of muscle (Ferreiro et al., 2002a,b).

MmD is a heterogeneous disorder with variable genetic causes and specific muscle-group involvement (Ferreiro et al., 2000, 2002a,b; Jungbluth et al., 2005). The classical phenotype includes axial muscle weakness, rigid spine, scoliosis and severe respiratory impairment, and is caused by recessive mutations in *SELENON* (Ferreiro et al., 2002a). A moderate form of MmD is characterized by slowly progressive weakness of axial muscles, pelvic girdle and hand muscles. This subtype is not normally associated with respiratory impairment or scoliosis and is caused by recessive mutations in *RYR1* (Ferreiro et al., 2002b). Other clinical presentations include weakness and hypotonia of the facial and neck muscles with ophthalmoplegia and pharyngolaryngeal involvement (Jungbluth et al., 2005), or antenatal onset and multiple joint contractures (arthrogryposis) (Ferreiro et al., 2000). A severe form of MmD with cardiac involvement, progressive weakness and poor prognosis has also been reported (Shuaib et al., 1988), caused by mutations in *MYH7* (Cullup et al., 2012). Finally, mutations in *MEGF10* may result in MmD with severe weakness, respiratory impairment, scoliosis and joint contractures (Boyden et al., 2012). As the genetic causes of MmD expand, it is likely that so will the clinical presentations.

**Fig. 1.**
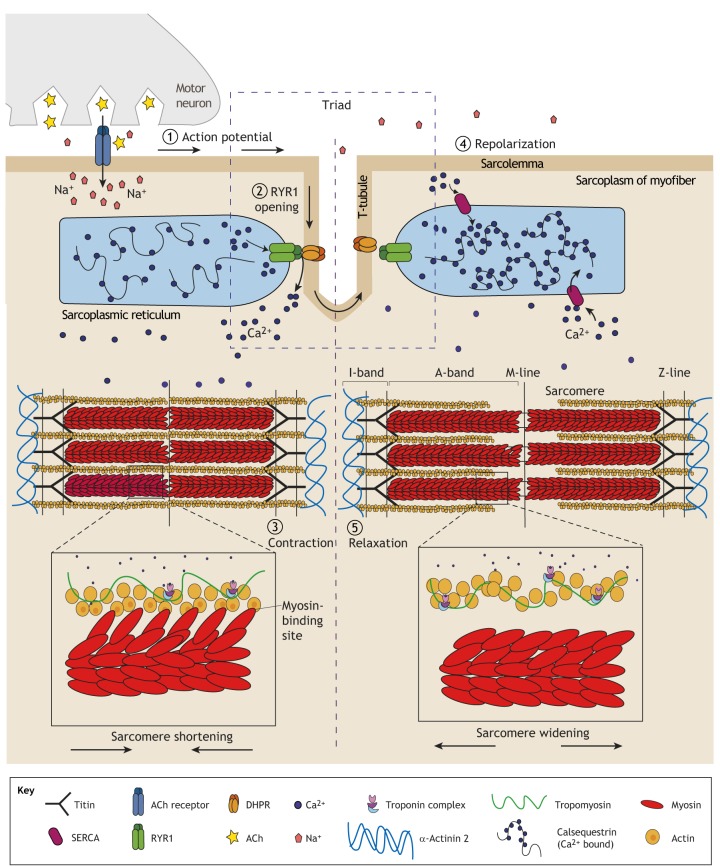
**Contraction and relaxation in skeletal muscle.** (1) In healthy skeletal muscle, an action potential from the motor neuron triggers acetylcholine (ACh) release at the neuromuscular junction, which induces an action potential along the muscle myofiber sarcolemma. The signal is propagated along the sarcolemma and the network of deep invaginations called T-tubules. T-tubules (shown here in dashed box) together with two terminal cisternae of the sarcoplasmic reticulum (SR), the main Ca^2+^-storage region in skeletal muscle, form the triad. The triad is central to excitation-contraction coupling (ECC), the process by which an action potential triggers the synchronous contraction of the myofibrils, leading to muscle contraction. (2) The change in membrane potential at the T-tubule caused by the action potential triggers a conformational change to the voltage-sensor subunit of the dihydropyridine receptor (DHPR), which triggers the opening of RYR1 in the terminal cisternae of the SR, to which it is mechanically coupled. RYR1 releases large amounts of Ca^2+^ into the sarcoplasm, where it interacts with the repeating contractile units of the myofibrils, called sarcomeres. (3) Ca^2+^ binds to the troponin complex, triggering the reconfiguration of the actin-tropomyosin structure, which exposes myosin-binding sites and allows myosin heads to bind to actin via crosslinks. Cyclical actin-myosin binding shortens the sarcomere via the sliding-filament mechanism first theorized by Huxley, Hansom and Niedergerke in 1954 (Huxley and Hanson, 1954; Huxley and Niedergerke, 1954). This results in muscle contraction. (4) Repolarization of the sarcolemma and T-tubules closes the DHPR and RYR1, preventing further Ca^2+^ release. Sarcoplasmic Ca^2+^ is rapidly sequestered into the SR via sarco/endoplasmic reticulum Ca^2+^-ATPase (SERCA) pumps, which enable the actin-tropomyosin structure to return to its original conformation, blocking myosin-head binding and resulting in muscle relaxation (5) (Gomes et al., 2002; Smith et al., 2016). See Box 3 for a glossary of certain terms.

**Fig. 2.**
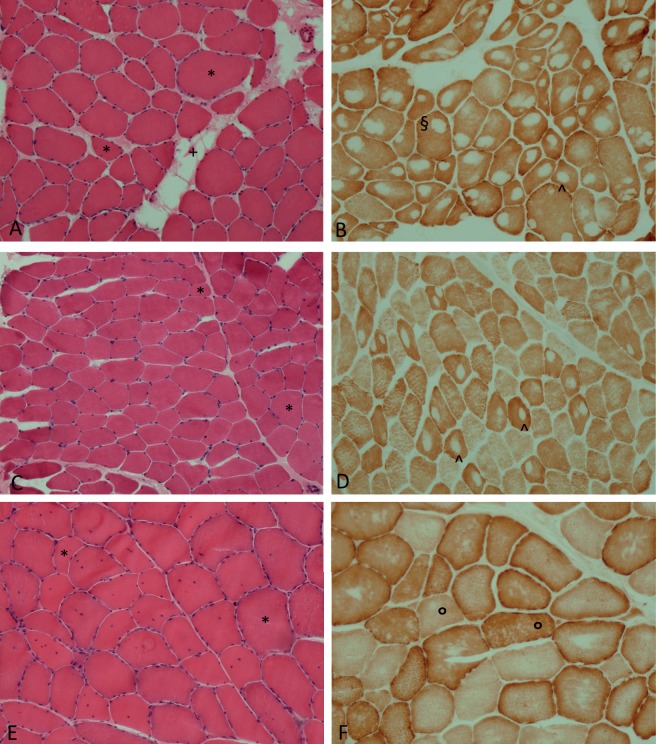
**Skeletal-muscle tissue sections from patients with core myopathy.** (A-D) Central core disease; (E,F) MmD. Samples range in age: 12 years (A,B), 28 years (C,D), 34 years (E,F). (A) Muscle shows myopathic features with fiber size variation and a mild increase in perimysial connective tissue with focal fatty infiltration and (B) a single central or eccentric core in most type 1 fibers. (C) Mild fiber size variation is shown. (D) Central cores are only present in the minority of type 1 fibers. (E) Myopathic features with fiber size variability and multiple central nuclei. (F) Multi-minicores in type 1 and type 2 fibers (Box 3) are shown. All patients carry *RYR1* mutations. *, fiber size variation; +, increase in perimysial connective tissue: ^, points to central core; §, eccentric core: °, multi-minicores. Image credit: E.P.

The genetic variants causing core myopathies primarily affect proteins involved in skeletal-muscle excitation-contraction coupling (ECC; see Box 3 for a glossary of terms; Fig. 1), either by altering calcium ion (Ca^2+^) transits between the sarcoplasmic reticulum (SR) and sarcoplasm (Box 3), or by disrupting the structure of the sarcomere (Box 3; Fig. 2). Ineffective ECC causes muscle weakness and is associated with the formation of mitochondria-depleted core lesions (Fig. 2). However, the processes governing core formation are far from completely understood. Furthermore, mutations in the proteins involved in ECC can give rise to a range of myopathic symptoms, which do not always result in core formation (Gonorazky et al., 2018). As next-generation sequencing further blurs the lines between genetic cause, histology and clinical phenotype, researchers have started to classify congenital myopathies based on the affected gene (e.g. ryanodine-related myopathies) or structure (e.g. triadopathies) (Lanner et al., 2010). However, as mutations in a single gene can result in multiple forms of congenital myopathy (with and without cores), it is likely that the protein dysfunction differs too.

**Box 3. Abbreviations and glossary**

**4-CmC:** 4-chloromethcathinone; a synthetic derivative of cathinone that induces RYR1-mediated Ca^2+^ release from the sarcoplasmic reticulum.

**[^3^H]-ryanodine binding assay:** this technique takes advantage of the highly specific binding of ryanodine to the open conformation of RYR1 to assess RYR1 kinetic properties.

**A-band:** a region of the sarcomere composed of thick myosin filaments (see Fig. 1 for details).

***ACTA1*:** skeletal alpha (α)-actin gene; it encodes a key component of skeletal muscle sarcomeres, the thin filaments.

***ACTN2*:** alpha (α)-actinin 2 gene; in sarcomeres, α-actinins are located in the Z-disc, where they anchor actin filaments.

**Bio-printing:** a technique that uses 3D printing technologies to combine cells, growth factors and various matrices (i.e. hydrogels, nanomaterials etc.) to maximally mimic tissues.

***CACNA1S*:** calcium voltage-gated channel subunit α1 gene; encodes the voltage-dependent L-type calcium channel α1S subunit [also known as dihydropyridine receptor (DHPR) and Ca_v_1.1].

**CASQ1:** calsequestrin 1; this skeletal-muscle-specific protein binds Ca^2+^ with high affinity and acts as a Ca^2+^ sensor within the sarcoplasmic reticulum.

***CCDC78*:** coiled-coil domain-containing protein 78 gene. CCDC78 is localized in the perinuclear region, sarcolemma membrane and T-tubule regions of the sarcoplasm. Mutations are associated with congenital myopathy, but the exact protein function(s) are currently unknown.

**CDK4:** cyclin-dependent kinase 4; a kinase involved in cell cycle regulation.

**CNFs:** centrally nucleated fibers; myofiber nuclei that form in a central, rather than peripheral, location; often a hallmark of regenerating fibers.

**CRU:** calcium release unit; this comprises junctional domains of the sarcoplasmic reticulum and the exterior membranes of the T-tubules, containing RYR1 and DHPRs, respectively. Release of Ca^2+^ from the CRU causes excitation-contraction coupling (ECC).

**DHPR:** dihydropyridine receptor (also called Ca_v_1.1); the protein, encoded by *CACNA1S*, is located on the T-tubule membrane and interacts with RYR1. An action potential alters the T-tubule membrane potential, changing the confirmation of DHPR, which opens RYR1, triggering intracellular Ca^2+^ release and muscle contraction.

**ECC:** excitation-contraction coupling. The process by which an action potential propagates along the sarcolemma and T-tubule invaginations, triggering Ca^2+^ release and causing the synchronous shortening of aligned sarcomeres and thus leading to muscle contraction.

**ER:** endoplasmic reticulum; a continuous membrane network within cells involved (amongst other roles) in protein and lipid synthesis and folding.

***ERO1*:** endoplasmic reticulum oxidoreductase 1 gene; catalyzes protein disulphide bond formation within the endoplasmic reticulum.

**Fluo-4:** one of several Ca^2+^ indicator dyes whose fluorescence increases upon binding to intracellular Ca^2+^. It is used to measure Ca^2+^ concentrations in living cells.

**FRET:** fluorescence resonance energy transfer; this technique measures whether two molecules interact with each other via the highly distance-dependent energy transfer between two light-sensitive molecules at the nanometer scale. It allows visualization at a subcellular and provides higher resolution compared to immunofluorescence.

**Fura-2:** the first Ca^2+^ indicator dye; increases in fluorescence upon binding to intracellular Ca^2+^ and is used to measure Ca^2+^ concentrations in living cells.

***FXR1*:** fragile X-related 1 gene; encodes an RNA-binding protein required for embryonic and postnatal development of muscle.

**GCaMP6:** a genetically encoded fluorescent Ca^2+^ indicator, consisting of green fluorescent protein (‘G’), calmodulin (‘Ca’) and calmodulin-interacting M13 peptide (‘MP’).

**HDAC:** histone deacetylase; a group of enzymes that, among other functions, regulate gene expression by removing acetyl groups from histones.

**HEK-293 cells:** an immortalized human embryonic kidney cell line widely used in cell and molecular biology research because of their reliable growth and propensity for transfection.

**hTERT:** human telomerase reverse transcriptase; an enzyme that lengthens telomeres. Forced expressed of hTERT prevents primary cells from undergoing replicative senescence, thereby immortalizing them.

**I-band:** an area of the sarcomere in which thin filaments (actin) are not superimposed by thick filaments (myosin) (Fig. 1).

**Kyphoscoliosis:** a spinal deformity characterized by abnormal curvature of the vertebral column along the coronal and sagittal planes. Combines kyphosis (convex curvature) and scoliosis (sideways curvature).

***MEGF10*:** multiple epidermal growth factor-like domains protein 10 gene; in skeletal muscle, MEGF10 regulates satellite cell proliferation and mutations are associated with MmD.

**MRI:** magnetic resonance imaging; a technique that uses magnetic fields and radio waves to image the structure of organs in 3D in a non-invasive manner.

***MYH7*:** myosin heavy chain 7 gene; it encodes for β-myosin heavy chain protein expressed in type 1 myofibers and cardiac cells. β-myosin heavy chain forms part of the type 2 myosin hexamer of sarcomeric thick filaments.

**Myofibril:** an aligned sarcomeric unit that extends along the length of each myofiber.

**Nemaline rods:** thread-like (from Greek ‘nema’) or rod-like protein aggregates within myofibers of patients with nemaline myopathies. These electron-dense structures are visible in muscle biopsies and stain red with the Gomori's trichrome staining.

***RYR1*:** ryanodine receptor 1 gene; the RYR1 protein is located on the sarcoplasmic reticulum and releases Ca^2+^ in response to an action potential.

**Sarcolemma:** myofiber plasma membrane (Fig. 1).

**Sarcomere:** the basic contractile unit of skeletal muscle myofibers; it is the repeating unit between two Z-lines that shortens with muscle contraction (Fig. 1).

**Sarcoplasm:** the cytoplasm of skeletal muscle cells (Fig. 1).

***SECISBP2*:** selenocysteine insertion sequence binding protein 2 gene.

***SELENON*:** alternative symbols are *SELN* and *SEPN1*; gene that encodes selenoprotein N, a glycoprotein of the sarco/endoplasmic reticulum involved in the regulation of redox-related Ca^2+^ homeostasis and myogenesis.

**SERCA:** sarco/endoplasmic reticulum Ca^2+^-ATPase; this ATPase transfers Ca^2+^ from the sarcoplasm to the lumen of the sarcoplasmic reticulum during muscle relaxation (Fig. 1).

**SR:** sarcoplasmic reticulum; the specialized endoplasmic reticulum of skeletal muscle and the main storage area of Ca^2+^ in myofibers (Fig. 1).

**T-antigen:** large antigen from simian virus 40. It has a role in viral genome replication and displays highly oncogenic activities by corrupting the host cell cycle checkpoints. It is often used to immortalize cell lines.

**Tetrad:** a structural DHPR/RYR1 link that allows ECC in skeletal muscle.

**Triad:** a structure within skeletal muscle cells composed of a T-tubule flanked by two terminal cisternae of the sarcoplasmic reticulum.

***TTN*:** gene encoding titin, a large protein involved in the maintenance of sarcomeric organization during contraction and in developing passive tension during muscle stretching.

**Type 1 and type 2 myofibers:** skeletal myofibers can be divided into subtypes based on their metabolism and expression of MyHC isoforms. Type 1/I myofibers are oxidative slow-twitch with a high resistance to fatigue, whereas type 2/II myofibers have a glycolytic fast-twitch profile. Type 2 myofibers are further subdivided into type 2A, 2B and 2X.

**Z-line:** also known as Z-disk; the border between aligned sarcomeres (Fig. 1). Z-line streaming refers to disrupted Z-line borders and loss of sarcomere integrity, which can occur in several muscle disorders.

Irrespective of classification, many questions surrounding the mechanisms driving core myopathy pathogenesis remain, such as: is there a common mechanism for core formation in skeletal muscle? Why do mutations in ryanodine receptor 1 (*RYR1*; Box 3) result in such a wide range of disorders? What are the pathological mechanisms of non-*RYR1* core myopathies? These queries need to be understood in order to develop much-needed therapeutic options for patients with CCD or MmD. To answer these and other questions, the development of functional and recapitulative models that represent the ever-expanding spectrum of core myopathies is imperative.

## Histopathology

The distinctive histopathological features of CCD and MmD (Table 1) differentiates them from other neuromuscular disorders. As the name suggests, core myopathy biopsies show amorphous cores in a variable number of myofibers (Hayashi et al., 1989). These cores are characterized by the absence of mitochondria, a reduction in glycogen granules, and absence of oxidative enzyme and phosphorylase activity (Dubowitz and Pearse, 1960). Both disorders are also associated with type 1 fiber predominance (Ferreiro et al., 2000; Maggi et al., 2013) (Table 1; Box 3). Electron microscopy analysis of CCD biopsies reveals abnormal sarcomere structures within core lesions, with contracted sarcomeres and split myofibrils that misalign with the peripheral myofibrils outside the cores. The architecture of SR:T-tubule complexes is distorted both within the cores and in peripheral myofibrils, with dramatic increases in SR and T-tubule networks (Box 3) (Hayashi et al., 1989).

**Table 1.**
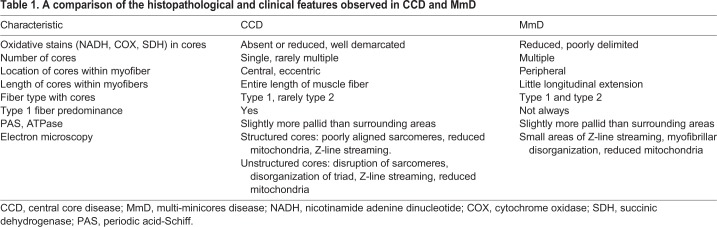
**A comparison of the histopathological and clinical features observed in CCD and MmD**

MmD cores are also mitochondria-poor regions. While CCD myofibers contain one or two cores that span the length of uniquely type 1 myofibers, MmD is defined by the presence of multiple small cores within both type 1 and 2 myofibers (Box 3) (Jungbluth et al., 2011). Moreover, centrally nucleated myofibers (CNFs; Box 3) are present in MmD subtypes (Ferreiro et al., 2000). Despite this, histopathological distinction of the core myopathies is not always clear, as muscle biopsies can show a mixture of cores, minicores and nemaline rods (Box 3) (Ferreiro et al., 2002b; Monnier et al., 2000; Scacheri et al., 2000).

## The genetic causes of core myopathies

**Fig. 3.**
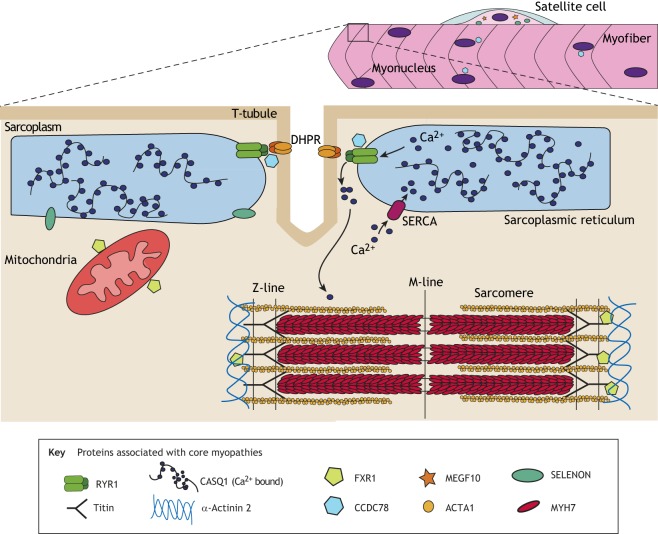
**The proteins currently identified as causing core myopathies, and their location in skeletal muscle.** Disease-associated proteins are identified in the Key. Schematic of a multinucleate myofiber with associated satellite cell, with a magnified region of the myofiber showing the excitation-contraction coupling (ECC) apparatus, sarcoplasmic reticulum (SR), sarcomere and mitochondria. CASQ1, calsequestrin 1; CCD78, coiled-coil domain-containing protein 78; DHPR, dihydropyridine receptor; FXR1, fragile X related 1; MEGF10, multiple epidermal growth factor-like domains protein 10; MYH7, myosin heavy chain gene; RYR1, ryanodine receptor 1; SELENON, selenoprotein N; SERCA, sarco/endoplasmic reticulum Ca^2+^-ATPase.

The relationship between the genetic defect and the resulting subtype of congenital myopathy is complex (reviewed in Gonorazky et al., 2018). Mutations in the same gene result in a variety of clinical and histopathological features within the congenital myopathy spectrum. Mutations in at least nine genes may result in core formation (Fig. 3).

### Ryanodine-related core myopathies

*RYR1* mutations are the most common cause of non-dystrophic congenital myopathies, with a US prevalence of 1/90,000 (Amburgey et al., 2011; Maggi et al., 2013; Savarese et al., 2016). There are over 300 known mutations in *RYR1* (Lanner et al., 2010) associated with CCD, MmD (non-classical type; Box 2), congenital fiber type disproportion, centronuclear myopathy (Wilmshurst et al., 2010), congenital muscular dystrophy (van Ruitenbeek et al., 2019) and core–nemaline-rod myopathy (Monnier et al., 2000). Moreover, *RYR1* mutations can also result in episodic manifestations of malignant hyperthermia syndrome (MHS; Box 4) (Treves et al., 2005), exertional rhabdomyolysis (Scalco et al., 2015) and periodic paralysis (Matthews et al., 2018). For an extensive review of *RYR1* function, see Lanner et al. (2010). The prevalence of RYR1-related core myopathies has been difficult to ascertain due to the wide range of causative mutations and clinical manifestations. Nevertheless, approximately 90% of CCD cases are caused by autosomal-dominant *RYR1* mutations, mostly localized in three hotspots: the cytoplasmic N-terminus [hotspot 1; amino acid (AA) 35-614], the central domain (hotspot 2; AA 2163-2458) and the C-terminus (hotspot 3; AA 4550-4940) (Jungbluth et al., 2011; Wu et al., 2006).

**Box 4. Focus on malignant hyperthermia syndrome (MHS)**

MHS is an autosomal-dominant pharmacogenetic disorder often associated with CCD and MmD that is caused by hyper-responsive RYR1-channel kinetics (McCarthy et al., 2000). Upon application of heat, muscle relaxants (e.g. succinylcholine) or inhaled anesthetics (e.g. halothane), susceptible individuals present with rapidly increased body temperature and heart rate, prolonged muscle contraction, respiratory acidosis, and rhabdomyolysis (Mickelson and Louis, 1996). If not treated quickly with the RYR1 antagonist dantrolene to lower intracellular Ca^2+^, it can be fatal (Lopez et al., 1987). Susceptibility to MHS is diagnosed by the standardized European *in vitro* contracture test, where a muscle biopsy is exposed to incremental doses of stimulants (halothane, caffeine, succinythane) to assess the threshold of muscle contraction (Cacheux et al., 2015; Gillard et al., 1992; Mickelson and Louis, 1996; Quane et al., 1993; Shuaib et al., 1987). If threshold contraction levels are lower than normal, the patient is deemed at risk of MHS. Although this test is invasive and can give false-positive and false-negative results (Mickelson and Louis, 1996), MHS guidelines still recommend the *in vitro* contracture test and molecular genetic screening for patient safety (Girard et al., 2004).

Defects in RYR1 alter ECC and Ca^2+^ homeostasis via two proposed pathological mechanisms: the ‘leaky channel’ and ‘EC uncoupling’. Mutations in hotspot 1 or 2 cause RYR1 to be ‘leaky’, decreasing the threshold for channel activation and leading to channel hyperactivity and precocious Ca^2+^ release from the SR (Box 3) (Avila and Dirksen, 2001; Treves et al., 2005). Patients carrying these mutations are often susceptible to malignant hyperthermia syndrome (MHS; Box 4) (McCarthy et al., 2000).

Mutations in hotspot 3 of *RYR1* are associated with the ‘EC uncoupling’ mechanism, which leads to a defective coupling between membrane depolarization and Ca^2+^ release from the SR (Avila et al., 2002; Treves et al., 2005). This may be due to dominant-negative suppression of RYR1-channel Ca^2+^ permeation (Loy et al., 2011). However, as genetic testing is expanding the range of *RYR1* mutations in core myopathies, it is becoming clear that the pathogenic mechanism cannot be predicted purely by mutational position. For example, several hotspot 2 mutations manifest with dual pathogenic characteristics: a RYR1 that is hypersensitive to agonist and voltage activation and, simultaneously, has increased basal activity (Dirksen and Avila, 2004).

Recessive mutations in *RYR1*, resulting in decreased protein expression, are seen in rare cases of CCD (Ducreux et al., 2006; Jungbluth et al., 2013; Wu et al., 2006). Diminished RYR1 expression is associated with a more severe phenotype (Amburgey et al., 2013), likely due to the critical role of the RYR1 in ECC. Recessive *RYR1* mutations also account for some non-classical forms of MmD (Jungbluth et al., 2005), and CCD that transiently presents as MmD with regards to muscle involvement and core lesions (Ferreiro et al., 2002b). In addition to protein loss, recessive mutations in *RYR1* also trigger a number of epigenetic perturbations in skeletal muscle: *RYR1* hypermethylation, increased class II HDAC (Box 3) expression, and reduction in muscle-specific miRNAs, which in turn further reduce RYR1 protein expression (Rokach et al., 2015).

### Other genetic causes

Around 50% of MmD cases, mostly classical (Jungbluth et al., 2018), are caused by mutation in *SELENON* (Box 3) (Ferreiro et al., 2002a), which encodes selenoprotein N. This glycoprotein regulates Ca^2+^ signaling and is involved in antioxidant pathways (Box 3) (Ferreiro et al., 2002a; Pitts and Hoffmann, 2018), and has a roles in embryogenesis (Castets et al., 2009) and myogenesis (Castets et al., 2011). A recent study by Bachmann and colleagues demonstrated that, like recessive *RYR1* mutations (Rokach et al., 2015; Zhou et al., 2006), mutations in *SELENON* lead to hypermethylation of genes involved in Ca^2+^ signaling (Bachmann et al., 2019), suggesting a potentially shared disease mechanism.

Other genes involved in MmD are *MYH7* (Cullup et al., 2012), *TTN* (Chauveau et al., 2014), *MEGF10* (Boyden et al., 2012; Takayama et al., 2016), *SECISBP2*, *ACTA1*, *ACTN2*, *CCD78* (Kazamel and Milone, 2019) and, recently, *FXR1* (Estañ et al., 2019) (Box 3). Mutations in the dihydropyridine receptor (DHPR) gene *CACNA1S* (Box 3) also results in the presence of cores in some families (Schartner et al., 2017). Despite this, many patients do not have a definitive genetic diagnosis (Maggi et al., 2013), and the genetic spectrum is expected to increase with increased uptake of genetic testing, increasing the need for accurate disease models to improve our understanding of the pathology and treatment options.

## *In vitro* models of core myopathies

*In vitro* culture affords the unique opportunity to deconstruct and reconstruct various aspects of disease pathogenesis in an effort to remove confounding parameters and hone in on the disease-causing mechanism. The study of core myopathies *in vitro* has employed various cell types derived from skeletal muscle (primary or immortalized myoblast progenitors that differentiate and fuse into multinucleate myotubes) and non-muscle (HEK-293 and B-cell; Box 3) tissues, each with intrinsic advantages and limitations (summarized in Table 2). The implementation of culture models to study CCD and MmD has enabled researchers to understand the role of *RYR1* in disease progression, as discussed below. Despite this progress, the pathogenic mechanisms underlying core myopathies remain unresolved. This is partially due to the difficulty in recapitulating the main pathological features – such as cores, altered Ca^2+^ handling and muscle weakness – in an *in vitro* environment, and emerges as a major challenge to mechanistic studies of CCD and MmD.

**Table 2.**
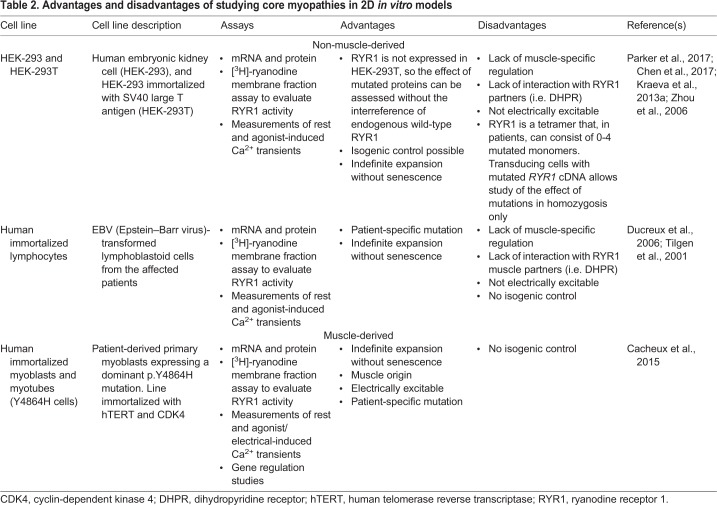
**Advantages and disadvantages of studying core myopathies in 2D *in vitro* models**

### RYR1 studies in culture

HEK-293 cells are of non-muscle origin and do not constitutively express RYR1. By comparing transiently expressed wild-type and patient *RYR1* variants, pathogenic mutations can be quickly identified using simple functional tests, such as the Ca^2+^ release assays (Parker et al., 2017). For example, Lynch, Tong and colleagues (Lynch et al., 1999; Tong et al., 1997) expressed patient *RYR1* cDNA in HEK-293 cells to assess the pathogenicity of several mutations using Fura-2 (Box 3), which enabled them to quantify RYR1-induced Ca^2+^ release.

*RYR1*-related cases of MmD are often associated with decreased RYR1 activity, which can be measured using the [^3^H]-ryanodine binding assay on membrane fractions (Box 3). This method is well-suited to culture studies and permits quantification of the functional consequences of different mutations (Zhou et al., 2006, 2010).

Despite many areas of RYR1 biology being well-suited to culture study, some functionalities are not. For example, non-muscle cell lines are not suitable for evaluating the effect of RYR1 variants on ECC, as this requires electrically excitable cells. Furthermore, cells lacking endogenous *RYR1* expression cannot recapitulate the complexity required to model the effect of dominant *RYR1* mutations on channel function. In skeletal muscle, RYR1 is arranged as a homotetrameric channel (Fig. 1) (Yan et al., 2015). Therefore, tetramers from individuals with *RYR1* mutations will consist of between zero and four mutant subunits in a mosaic pattern of variable functionality, as theorized in MacLennan and Zvaritch (2011). Without wild-type RYR1, the full range of tetramer combinations is challenging to replicate and can result in different phenotypes. Moreover, modeling dominant *RYR1* mutations in RYR1-expressing cells does not always recapitulate the phenotype; Kraeva and colleagues found that altered Ca^2+^ release was only observed when patient *RYR1* was expressed in *Ryr1*^−/−^ myotubes and not in *Ryr1*^+/−^ ones, despite the fact that the patient mutation (*RYR1* p.R4892Q) was dominant (Kraeva et al., 2013a). The authors concluded that *in vitro* modeling itself may further alter phenotype.

Combined, these studies show that 2D cell cultures can be extremely useful to confirm the pathogenicity of novel core myopathy mutations and provide initial phenotypic assessment, but functional effects should be interpreted with caution.

### Quantifying Ca^2+^ handling

Ca^2+^ signaling is critical to many cellular processes and therefore must be carefully regulated. The SR is the main store of Ca^2+^ in myofibers, but mitochondria and lysosomes also participate in Ca^2+^ homeostasis (Raffaello et al., 2016). Given the central role of Ca^2+^ handling in the pathogenesis of core myopathies, the ability to observe intracellular ion fluxes is fundamental. Some aspects of myofiber Ca^2+^ handling cannot currently be studied in culture due to improper subcellular compartmentalization, as myofiber maturation *in vitro* is limited. Despite this, most Ca^2+^ detection methods are best suited to analysis in culture, providing a unique opportunity to relate disease mutations to Ca^2+^ handling defects. Ca^2+^-binding fluorescent dyes, such as Fura-2AM and Fluo-4 (Box 3), allow researchers to observe Ca^2+^ handling at rest and in response to RYR1-opening stimuli (Kraeva et al., 2013a; Parker et al., 2017). The main limitation of cytosolic Ca^2+^-binding fluorescent dyes is that they do not discern the source of the ion, i.e. whether it originated from the SR. A FRET-based (Box 3) imaging approach with the Ca^2+^-sensitive chameleon protein D1ER (Palmer et al., 2004) can increase resolution and sensitivity. This ER (Box 3)-targeted protein enables one to specifically visualize Ca^2+^ storage capacity and depletion in the ER (SR in skeletal muscle) compared to other Ca^2+^ storage regions in the cell (Chen et al., 2017), with fluorescence intensity directly correlated to Ca^2+^ binding. FRET is particularly well-suited to 2D culture since cells can be grown on surfaces that are amenable to high-resolution microscopy. However, FRET is currently a low-throughput technique and can be challenging for non-experts, limiting its widespread adoption for the study of Ca^2+^ handling.

### Myofiber maturation in culture

A major challenge for *in vitro* studies of core myopathies is the limited structural maturation of myotubes in 2D culture, which develop only some of the structures that can be found in myofibers *in vivo* (Cooper et al., 2004; Cusimano et al., 2009; Flucher et al., 1991). This is true for both primary (embryonic and adult) and immortalized myoblast-derived myotubes. In general, 2D culture substrates are ill-suited to support the long-term maintenance of contractile myofibers, resulting in the loss of the most mature cells following their contraction and enrichment for more immature myofibers over time (Afshar Bakooshli et al., 2019; Guo et al., 2014). This can be overcome by modifying the composition of the culture media (Guo et al., 2014), for example by including a nerve-derived factor, or through the use of a reconstituted basement-membrane-like overlay atop the developing myotubes (Falcone et al., 2014; Pimentel et al., 2017).

Typical 2D-cultured myotubes also have different transcriptomic profiles than those in adult animals, especially concerning genes involved in ECC. This problem was highlighted by Bachmann et al. (2019), who found that expression of *RYR1*, *CACNA1S* and *ATP2A1* was decreased in patient muscle biopsies, but increased in the primary myotube cultures obtained from those patients’ myoblasts. Epigenetic status is tissue specific and can also be affected by skeletal-muscle maturation stage and architecture (Attali et al., 2013; Rokach et al., 2015). Indeed, differences in the expression of epigenetic modifiers have also been observed between patient biopsies and 2D-cultured myotubes derived from the patient’s primary myoblasts (Bachmann et al., 2019). These limitations may explain why it is challenging to specifically model core myopathies in culture and highlight the need to identify biochemical and biophysical cues that can push maturation of cultured myotubes. Research in this direction may, along the way, provide insights into disease pathogenesis (see 3D human cell culture models section). Nevertheless, 2D myofiber cultures have uncovered the pathological consequences of several core myopathy gene mutations (Castets et al., 2009, 2011; Estañ et al., 2019), and *in vitro* myofiber cultures are especially useful as preclinical models to test potential therapeutic interventions in human cells and when animal models are not available.

## Animal models of core myopathies

Upon establishing a genetic cause for *RYR1*-related core myopathy (Quane et al., 1993), researchers developed transgenic animals, enabling the study of protein function and pathogenic consequences – both in the muscle and in other tissues – in an intact, representative system. The most common animal models vary from loss-of-function models to patient-specific mutations in *RYR1*. Here, we outline animal models presenting with a skeletal-muscle myopathy, and identify their uses and phenotypic overlap with the human condition (summarized in Table 3). As such, *Ryr1^S2844D^* mice (Andersson et al., 2011) and the recent MHS *Ryr1^pG2435R^* mice (Lopez et al., 2018) are not discussed. Excellent reviews of RYR models, including *Ryr2* mutants, can be found elsewhere (Betzenhauser and Marks, 2010; Hanna et al., 2016; Kushnir et al., 2010).

**Table 3.**
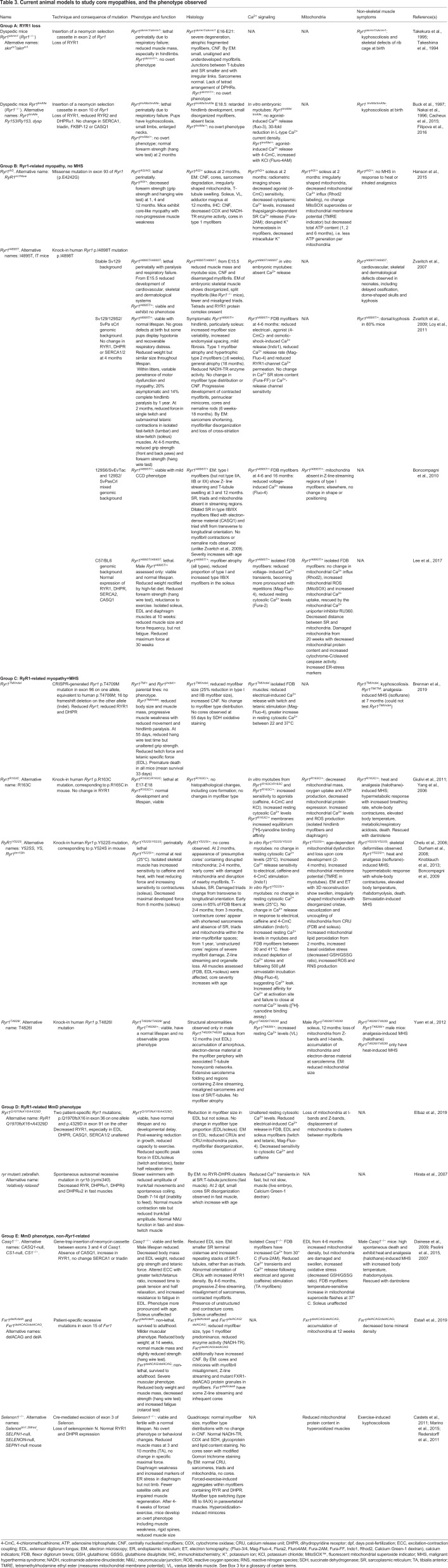
**Current animal models to study core myopathies, and the phenotype observed**

### RYR1-related core myopathies

As mentioned above, some of the most severe cases of core myopathy are caused by reduced *RYR1* expression (Amburgey et al., 2013). The requirement of RYR1 for skeletal-muscle function was established using two *Ryr1-*knockout mouse strains in the 1990s. *Ryr1^skrrm1^*, established by Takeshima and colleagues, contains a mutation at exon 2 of murine *Ryr1* (Takeshima et al., 1994), whereas *Ryr1^tmAll^*^e^ contains an insertion at exon 10 (Buck et al., 1997). Homozygous *Ryr1^−/−^* mice are often referred to as dyspedic as they lack the cytoplasmic ‘foot’ domain that anchors RYR1 to the SR (Takekura et al., 1995; Takeshima et al., 1994). *Ryr1^−/−^* mice from both backgrounds die perinatally of respiratory failure due to a lack of ECC, have severely reduced muscle mass and have skeletal abnormalities (Hanson et al., 2015; Takekura et al., 1995; Takeshima et al., 1994). Indeed, impaired Ca^2+^ release following depolarization in isolated neonatal muscles abolished their response to electrical stimulation (Takeshima et al., 1994). Neonatal *Ryr1^−/−^* myofibers are small with underdeveloped myofibrils and lack RYR1 and tetrads (Box 3) (Takekura et al., 1995). Despite reduced *Ryr1* levels, heterozygous *Ryr1^skrrm1/+^* and *Ryr^tmAll^*^e*/+*^ mice have no reported physiological or histopathological abnormalities (Takekura et al., 1995; Takeshima et al., 1994). This appears to correlate with human pathology; RYR1-associated core disease is caused by autosomal-dominant mutations or biallelic recessive *RYR1* loss, rather than heterozygous loss. Nevertheless, this somewhat limits the use of *Ryr1^+/−^* mice for core myopathy research.

Several *RYR1* knock-in transgenic models have been developed with patient-specific mutations. These mice show considerable strain variability (Table 3), which has provided great insight into the pathological mechanisms governing different *RYR1* mutations. There are currently two knock-in lines with N-terminal *Ryr1* mutations resulting in a core myopathy with MHS (Box 4). *Ryr1^Y522S/+^* (Chelu et al., 2006) and the milder *Ryr1^R163C^* (Yang et al., 2006) mice exhibit heat- and analgesia-induced MHS, resulting in a hypermetabolic state with increased body temperature, whole-body contractions and death (Chelu et al., 2006; Durham et al., 2008; Yang et al., 2006). Both mutations increase RYR1-channel sensitivity to electrical and agonist stimulation at physiological temperatures, resulting in SR Ca^2+^ leakiness and store depletion (Chelu et al., 2006; Durham et al., 2008; Knoblauch et al., 2013; Yang et al., 2006). *Ryr1^Y522S/+^* mice also have increased sensitivity to the statin simvastatin, which is a useful consideration for clinicians if prescribing statins to core myopathy patients (Knoblauch et al., 2013).

Ryanodine-binding assays showed that Ca^2+^ leakiness in these mice is due to an increased Ca^2+^-binding affinity in mutant channels and failure of Y522S RYR1 channels to close at normal Ca^2+^ levels (Durham et al., 2008; Yang et al., 2006). This results in dysfunctional mitochondria, increased levels of reactive oxygen species (ROS) and reactive nitrogen species (RNS) (causing oxidative/nitrosative stress), and decreased maximal developed muscle force in older *Ryr1^Y522S/+^* mice (Durham et al., 2008; Giulivi et al., 2011). While the Y522S and R163C mutations cause CCD in humans (Quane et al., 1993), initial studies did not detect cores in *Ryr1^Y522S/+^* or *Ryr1^R163C/+^* mice (Chelu et al., 2006; Durham et al., 2008). However, a careful temporal analysis by Boncompagni and colleagues showed that damaged mitochondria in *Ryr1^Y522S/+^* mice localize in specific subcellular regions of myofibers, which they termed ‘presumptive cores’ (Boncompagni et al., 2009). Localized regions with damaged mitochondria are associated with disrupted sarcomeres, triads and T-tubules and/or SR, which over time become progressive cores like those observed in patients (Box 3). Thus, the authors proposed the first mechanism for mutation-specific core development whereby SR Ca^2+^ leakage causes oxidative damage, which disrupts local mitochondria and the sarco-tubular system. Damage and eventual loss of mitochondria and SR prevents Ca^2+^ reuptake, and high Ca^2+^ levels cause prolonged sarcomere contraction (contracture cores), which activates proteolysis and leads to sarcomere degeneration (unstructured cores) (Boncompagni et al., 2009).

Additionally, three mouse lines with C-terminal *Ryr1* mutations develop phenotypes that do not all align with patient symptoms. The T4826I mutation in the transmembrane domain of *RYR1* is associated with MHS without core myopathy in humans (Brown et al., 2000). However, *Ryr1^T4826I/T4826I^* knock-in mice have increased resting Ca^2+^ levels and aged male *Ryr1^T4826I/T4826I^* mice show ultrastructural core-myopathy-like features in the soleus (Box 3), including Z-line streaming (Box 3), misaligned sarcomeres and loss of myofibrillar mitochondria (Yuen et al., 2012).

The *Ryr1^I4895T^* mouse carries the severe I4898T CCD mutation (Lynch et al., 1999; Tilgen et al., 2001; Zvaritch et al., 2009). Whereas the R163C and Y522S mutations cause ‘leaky’ Ca^2+^ receptors, the I4898T mutation in the C-terminal transmembrane RYR1 domain results in an ‘uncoupled’ phenotype. Similar to other dominant mutations, homozygous *Ryr1^I4895T/I4895T^* mice die of respiratory failure at birth, with delayed musculoskeletal, cardiac and dermatological development (Zvaritch et al., 2007). *Ryr1^I4895T/+^* mice develop normally, although some neonate pups have recoverable hypotonia and respiratory distress (Zvaritch et al., 2009). With age, *Ryr1^I4895T/+^* mice exhibit a progressive myopathy with muscle weakness, atrophic type 1 myofibers with enzyme-poor perinuclear cores, and some mice also develop minicores and nemaline rods (Box 3) (Zvaritch et al., 2009). Ultrastructural analyses reveal abnormal myofibrillar organization and a phenotype consistent with premature muscle aging (Boncompagni et al., 2010). Unlike CCD, no type 1 fiber predominance is observed in *Ryr1^I4895T/+^* mice (Lee et al., 2017; Zvaritch et al., 2009). Despite intact RYR1 Ca^2+^ release units (CRUs; Box 3) and preserved SR Ca^2+^ content, *Ryr1^I4895T/I4895T^* myofiber cultures have dramatically reduced stimulant-induced Ca^2+^ release (Zvaritch et al., 2007), and intact *Ryr1^I4895T/+^* muscles have decreased resting cytosolic Ca^2+^ levels and Ca^2+^ release (Lee et al., 2017). This occurs due to reduced Ca^2+^-channel sensitivity from a mutation-induced RYR1 pore blockade. Partially functional wild-type:mutant RYR1 heterotetramers in *Ryr1^I4895T/+^* mice allow some Ca^2+^ release and force generation, with severity depending on tetramer ratios (Lee et al., 2017; Loy et al., 2011; Zvaritch et al., 2009).

An interesting observation from *Ryr1^I4895T/+^* mice is that the pathological phenotype depends on the genetic background: whilst *Ryr1^I4895T/+^* on a Sv129 background have no overt phenotype (Zvaritch et al., 2007) and C57BL/6 *Ryr1^I4895T/+^* myopathy is mild (Lee et al., 2017), *Ryr1^I4895T/+^* mice on mixed genetic backgrounds exhibit a highly variable, progressive myopathy (Boncompagni et al., 2010; Zvaritch et al., 2009). Indeed, motor dysfunction between littermates varies from asymptomatic to complete limb paralysis by 12 months (Zvaritch et al., 2009). Moreover, this phenotype varies between different mixed backgrounds, with nemaline rods and myofibril contractures observed by some (Zvaritch et al., 2009), but not by others (Boncompagni et al., 2010). This mimics the well-documented variable penetrance in patients harboring this mutation (Jungbluth, 2007; Lynch et al., 1999; Tilgen et al., 2001). However, the progressive symptoms and presence of minicores, cores and rods in mixed-background *Ryr1^I4895T/+^* mice has led to the speculation that these strains do not recapitulate human probands as well as stable lines do (Lee et al., 2017; Lynch et al., 1999; Zvaritch et al., 2009). Interestingly, genetic background also modulates the phenotype of other modeled neurological diseases, including dystonia (Tanabe et al., 2012). A potential reason for this is that genetic modifiers with protective/permissive roles differ between strains, affecting disease penetrance. Mapping these in mice and humans would provide insight into the molecular pathways governing disease progression.

An alternative model is the *Ryr1^AG^* strain, which contains a p.E4242G missense mutation in exon 93, has a stable a non-progressive core-like myopathic phenotype with decreased muscle strength, disrupted Ca^2+^ signaling and abnormal mitochondrial function (Hanson et al., 2015). While the mutation is not directly linked to a patient variant, this strain provides a well-characterized model in which to test potential therapies for C-terminal *RYR1* mutations.

### Recessive *Ryr1* models

Two new models of recessive *RYR1*-related myopathies have recently been developed (Brennan et al., 2019; Elbaz et al., 2019) that aim to recapitulate the severe pediatric phenotypes of a subset of *RYR1*-related core myopathy patients (Amburgey et al., 2013; Jungbluth et al., 2011). The *Ryr1^TM/Inde^* strain contains the patient point mutation T4706M on one allele and a frameshift deletion on the other *Ryr1* allele (Brennan et al., 2019). These mice have smaller muscles and exhibit severe, rapidly progressive muscle weakness resulting in premature death. Atrophic myofibers are weaker and have reduced force and electrical-induced Ca^2+^ release, likely due to decreased RYR1 and DHPR (Brennan et al., 2019). The parental *Ryr1^TM/TM^* line also exhibits MHS, as do patients carrying the same mutation (Amburgey et al., 2013; Jungbluth et al., 2011). However, unlike patients, *Ryr1^TM/Inde^* mice do not develop cores/minicores or type 1 fiber predominance, two features of recessive *RYR1* mutations, and, unlike patients, the mice have increased CNFs (Amburgey et al., 2013; Clarke et al., 2010). The *Ryr1^Q1970fsX16/A4329D^* strain also contains biallelic mutations, a frameshift and a missense, as reported in severe MmD cases (Zhou et al., 2007). *Ryr1^Q1970fsX16/A4329D^* mice recapitulate patient symptoms, including reduced growth, muscle weakness, decreased Ca^2+^ transients, loss of RYR1, and ultrastructural changes such as reduced CRUs and intermyofibrillar mitochondria, misaligned myofibers and core-like lesions (Elbaz et al., 2019). Despite this, *Ryr1^Q1970fsX16/A4329D^* mice have a less-severe phenotype than *Ryr1^TM/Inde^*, with no reduction in lifespan. Whilst the *Ryr1^TM/Inde^* and *Ryr1^Q1970fsX16/A4329D^* lines are interesting new models that fill an unmet need to study severe recessive *RYR1*-related core myopathies, the mechanisms of disease progression are yet to be determined.

### Non-ryanodine mutations

*RYR1* mutations are the major cause of core myopathies and, as such, have been the most widely studied core myopathy mutation in animal models. Nevertheless, MmD is also caused by mutations in *SELENON* (Ferreiro et al., 2002a; Kazamel and Milone, 2019), *MYH7* (Cullup et al., 2012), *ACTA1* (Kaindl, 2004), *ACTN2* (Lornage et al., 2019), *TTN* (Chauveau et al., 2014), *MEGF10* (Boyden et al., 2012; Takayama et al., 2016), *CCDC78* (Kazamel and Milone, 2019) and *FXR1* (Estañ et al., 2019) (Box 3), prompting the development of several non-ryanodine core myopathy models.

As many as 50% of patients with classical MmD have recessive mutations in *SELENON* (Ferreiro et al., 2002a), and, in addition to enzyme-deficient minicores, their myofibers have excessive oxidative/nitrosative stress and abnormal Ca^2+^ handling (Arbogast et al., 2009). Although *Selenon1^−/−^* mice do not exhibit an overtly myopathic phenotype under normal conditions (Rederstorff et al., 2011), they have proved extremely useful in identifying dual pathomechanisms that may underlie disease progression. Firstly, selenoprotein N regulates Ca^2+^ levels in the ER/SR via SERCA2 activation (Marino et al., 2015). *Selenon1^−/−^* mice have a dysfunctional ER-stress response, as redox-regulating SERCA2 activity is inhibited, preventing Ca^2+^ uptake and leading to persistently high oxidative stress (Marino et al., 2015; Pozzer et al., 2019). High muscle activity exacerbates this, which may explain why muscle weakness is seen only in the diaphragm or upon forced exercise regimes (Pozzer et al., 2019; Rederstorff et al., 2011). Indeed, hyper-oxidization of unaffected muscles via *ERO1* (Box 3) overexpression dramatically increased the myopathic phenotype, reducing mitochondrial counts and resulting in the development of minicores (Marino et al., 2015). Secondly, both *Selenon1^−/−^* mice and patients with selenoprotein N deficiencies have reduced satellite cell numbers and, when challenged with injury, *Selenon1^−/−^* mice have impaired muscle regeneration (Castets et al., 2011). Together, these data suggest that persistent ER stress and high cytosolic Ca^2+^ levels caused by selenoprotein N loss impairs ECC and causes muscle weakness, which may progress further due to impaired regeneration.

CASQ1 is the main Ca^2+^-binding and -buffering protein in skeletal-muscle SR (MacLennan and Wong, 1971) (Box 3). It may also modulate RYR1 activity, although this is contentious (Beard et al., 2002; MacLennan and Zvaritch, 2011). In humans, *CASQ1* mutations are associated with a vacuolar myopathy with muscle weakness, SR protein aggregates (D'Adamo et al., 2016; Rossi et al., 2014; Semplicini et al., 2018) and MHS caused by altered Ca^2+^ release kinetics (Kraeva et al., 2013b). *Casq1^−/−^* mice have decreased Ca^2+^ transients, abnormal CRUs, atrophic myofibers and muscle weakness (Paolini et al., 2007, 2015). They also undergo a hypermetabolic MHS reaction when challenged with sensitizers (Dainese et al., 2009). Although a direct link to core myopathy has not been made in humans, older *Casq1^−/−^* mice develop progressive core-like structures (Paolini et al., 2015). Interestingly, the phenotype affects predominantly fast muscles [extensor digitorum longus (EDL) muscle but not soleus]. The phenotypes of *Casq1^−/−^* and *Ryr1*-mutant mice overlap strikingly (see Table 3; Canato et al., 2015), including increased mitochondrial damage and elevated ROS levels that can be ameliorated with antioxidant administration (Durham et al., 2008; Paolini et al., 2015). Of note, electron-dense aggregates (presumed by the authors to be CASQ1) were observed by electron-microscopy analysis of *Ryr1^I4895T/+^* mice (Boncompagni et al., 2010), further highlighting the overlapping phenotypes of congenital myopathies.

As next-generation sequencing expands the spectrum of core-myopathy-causing genetic variants, new animal models continue to be generated. These descriptive studies identify phenotypic overlap and open the possibility for characterization and therapeutic targeting of gene-specific pathomechanisms. For example, recessive mutations in exon 15 of *FXR1* were identified in two families with congenital myopathies of varying severity, exhibiting type 1 fiber predominance, Z-line streaming, CNFs and minicores (Estañ et al., 2019). Interestingly, CRISPR-generated mice containing the familial mutations, namely *Fxr1^delA/delA^* and *Fxr1^delACAG/delACAG^*, strikingly recapitulate the severity of the human probands. An alternative strategy to model *ACTN2*-related (Box 1) core myopathy, also called multiple structured core disease, was recently employed by Lornage et al., who transduced zebrafish and murine muscles with mutant *p.Leu727Arg Actn2* (Lornage et al., 2019). Within 4 weeks, mice exhibited muscle weakness and structural changes similar to human probands, including abnormal Z-lines and multiple structured cores (Lornage et al., 2019). While useful to prove causation, this approach did not recapitulate the non-muscle features of *ACTN2*-related myopathy such as kyphoscoliosis (Box 3) (Lornage et al., 2019).

A challenge in developing animal models of rare core myopathies is that their causative mutations are often associated with other congenital myopathies. For example, autosomal-dominant *CCDC78* mutations have been diagnosed as both centronuclear myopathy and MmD, as biopsies have internal nuclei with some patients also showing atypical cores (Majczenko et al., 2012). Further, minicores are observed in some families with recessive *MEGF10* mutations (Boyden et al., 2012). While mutant *ccdc78* and *meg10* zebrafish have abnormal musculature and impaired swimming, they do not have cores (Boyden et al., 2012; Majczenko et al., 2012), unlike zebrafish with *ryr1b* loss (Hirata et al., 2007). Finally, there are currently no models for *MYH7*, *TTN* or *ACTA1* mutations, which cause a subset of MmD with cardiac involvement (Chauveau et al., 2014; Cullup et al., 2012; Kaindl, 2004). However, *Drosophila* with Laing-distal-myopathy-associated L1729del *myh7* mutations develop myofibril disruption, cores and mitochondrial abnormalities, further blurring the genotype-phenotype boundaries (Dahl-Halvarsson et al., 2018).

### Insights from core myopathy models

Taken together, the *Ryr1^I4895T/+^*, *Ryr1^Y522S/+^*, *Ryr1^R163C^*, *Ryr1^Q1970fsX16/A4329D^* and *Ryr1^TM/Inde^* mice show striking phenotypic similarities to the histopathology, Ca^2+^ transients, ECC and muscle weakness of *RYR1*-related core myopathy patients (Jungbluth, 2007). These mice provide important insights into disease progression that could not be ascertained from human biopsies. For example, the theory that cores, minicores and rods derive from a common mechanism of focal shearing due to RYR1-tetramer heterogeneity was inferred in aging mice (Zvaritch et al., 2009). Further, unlike *in vitro* models, the mature skeletal muscle in animal models enables evaluation of protein dysfunction in a tissue that contains all the structures and protein complexes required for normal ECC. They also provide a model to assess non-muscle pathology, a severely understudied topic.

Notably, the range of mutations affecting different functional domains enables researchers to assess novel therapeutic strategies in a mutation-specific manner. Mitochondrial dysfunction – a hallmark of core myopathies – also occurs in a mutation-specific manner. While mitochondrial swelling, uncoupling from triads, dysfunction and eventual loss occur in many mouse models (Table 3), studies also revealed alternate mechanisms of damage. For example, whilst increased levels of mitochondrial ROS cause damage in *Ryr1^Y522S/+^* mice (Durham et al., 2008), *Ryr1^AG/+^* mice have unaltered mitochondrial ROS but reduced ATP content (Hanson et al., 2015). Furthermore, whilst increased mitochondrial ROS were thought to be due to ‘leaky Ca^2+^’ mutations (Andersson et al., 2011), Ca^2+^-induced mitochondrial ROS elevation has also been observed in *Ryr1^I4895T/+^* mice, despite their reduced cytosolic Ca^2+^ and their blocked (‘uncoupled’) channel mutation (Lee et al., 2017). Here, mutation-induced ER stress drives persistently elevated mitochondrial Ca^2+^ uptake, causing mitochondrial damage. Moreover, the authors targeted elevated ER stress in *Ryr1^I4895T/+^* mice with the chemical chaperone sodium phenylbutyrate, which significantly increased mitochondrial function, myofiber size and force generation (Lee et al., 2017). Whether ER stress is a significant feature of other ‘uncoupled’ *Ryr1* models (e.g. *Ryr1^AG/+^*) is yet to be determined.

In another therapeutic strategy, Hanson and colleagues showed that potassium (K^+^) supplementation or K^+^-channel inhibition rescued abnormal K^+^ homeostasis in *Ryr1^AG/+^* mice, thus improving the phenotype (Hanson et al., 2015). Finally, several groups have targeted increased ROS and mitochondrial damage due to Ca^2+^ leakage from the SR in *Ryr1^Y522S/+^* mice with the antioxidant N-acetylcysteine (NAC). In the first study, 8 weeks of 1% w/v NAC in drinking water protected against mitochondrial damage and force degeneration (Durham et al., 2008). Prolonged treatment led to reduced core formation, reduced levels of creatine kinase/lactate dehydrogenase and improved muscle strength, even when administered after symptoms appeared (Michelucci et al., 2017). Importantly, the pathology-reducing effects of NAC have been confirmed in the *relatively relaxed* zebrafish MmD model and in human myotubes (Dowling et al., 2012). Although several candidate pathways are activated in patient biopsies (Hanson et al., 2015), to our knowledge the only therapeutic intervention tested in human cells is targeting ROS with NAC (Dowling et al., 2012), which has led to a clinical trial in *RYR1*-related myopathy (NCT02362425).

## Knowledge gaps

The ever-expanding range of genetically engineered animals and 2D *in vitro* models outlined above is rapidly increasing our understanding of the core myopathies. Despite this, several current knowledge gaps need to be filled to develop treatments. On this cusp of disease knowledge, it is crucial to define the unanswered question and identify the most clinically relevant model in which to study it. Below, we speculate on some of these questions.

### Mechanism of core formation

Ever since the first observation of cores, researchers have aimed to identify the mechanism leading to their formation. Since the 1970s, it has been suggested that cores, minicores and rods have a common origin, as some muscle biopsies from the same patient, carrying mutant *RYR1*, show multiple lesions (Bethlem et al., 1978; Ferreiro et al., 2002b; Monnier et al., 2000; Scacheri et al., 2000). However, a major limitation in the study of core development is that human biopsies are taken once clinical symptoms (and thus cores) have appeared, making animal models crucial. Indeed, Boncompagni and colleagues performed one of the few studies on core development, with a histopathological analysis of *Ryr1^Y522S/+^* mice over a series of time points as cores formed. This work supported the notion that a single mutation can cause cores and rods in skeletal muscle, with rods appearing with age. The authors proposed a mechanism by which Ca^2+^ leak from the SR disrupts mitochondria and SR in areas of the myofibers, leading to a lack of Ca^2+^ sequestration and degradation of the contractile units, resulting in core formation. They also suggested that lesions do not form across the whole myofiber due to the stochastic expression of *RYR1* from the healthy or mutant allele within discrete regions of the myofiber (i.e. dominant heterozygous mutations), or due to heterogeneity in relative mitochondrial oxidative capacity (Boncompagni et al., 2009).

While this work provides important insights on disease progression, an understanding of the molecular processes by which the cores arise is yet to be determined, and whether these mechanisms occur in human muscle is yet to be confirmed. Furthermore, no studies have explained core formation caused by mutations in other genes. Current evidence suggests that altered Ca^2+^ transients are involved both in muscle weakness (Treves et al., 2005) and in core formation (Boncompagni et al., 2009). As such, it remains unclear why cores form in MmD cases caused by mutations in genes not involved in Ca^2+^ equilibrium, including *TTN* (Chauveau et al., 2014), *MYH7* (Cullup et al., 2012), *MEGF10* (Boyden et al., 2012; Takayama et al., 2016), *SECISBP2*, *ACTA1*, *ACTN2*, *CCDC78* (Kazamel and Milone, 2019) and *FXR1* (Estañ et al., 2019).

### *RYR1* genotype-phenotype discordance

Another long-standing quandary are the seemingly opposing phenotypes arising from mutations in *RYR1*. A major theory tested in cell models (Avila et al., 2002; Lynch et al., 1999) and transgenic mice (e.g. Chelu et al., 2006; Yang et al., 2006; Zvaritch et al., 2009) is that the disease phenotype depends on whether the mutation causes ‘leaky’ or ‘uncoupled’ RYR1 channels. *Ryr1^R163C/+^* and *Ryr1^Y522S/+^* mice have ‘leaky’ mutations, which are associated with MHS (Chelu et al., 2006; Yang et al., 2006). Conversely, the C-terminal mutation in *Ryr1^I4898T/+^* mice causes uncoupled RYR1 functionality (Avila et al., 2002; Lynch et al., 1999) that results in a severe core myopathy (Zvaritch et al., 2009). It has been suggested that mutations across different regions of *RYR1* can result in the same phenotype (e.g. ‘leaky’) due to the 3D structure of RYR1 and its interaction with other proteins (reviewed in Witherspoon and Meilleur, 2016). However, as previously outlined, there is not always a clear genotype-phenotype correlation between patients with the same mutation, and it is becoming clear that not all *RYR1* mutations fit into these two categories.

In addition to skeletal-muscle symptoms, individuals with core myopathies exhibit several non-muscle abnormalities, most commonly skeletal defects (reviewed in Jungbluth, 2007). Interestingly, RYRs are involved in signal transduction in osteoclasts and nerves (reviewed in Lanner et al., 2010). This may explain why RYR mutations can result in skeletal and nerve abnormalities such as kyphoscoliosis and periodic paralysis (Matthews et al., 2018). Furthermore, vascular abnormalities, including increased bleeding times, have been reported in some core myopathy patients (Lopez et al., 2016). This feature also was shown in *Ryr1^Y522S/+^* mice, and was reversed by dantrolene (Lopez et al., 2016), suggesting a role for RYR1 in smooth-muscle function (reviewed in Lanner et al., 2010). To develop future therapies that fully address the symptoms of core myopathies, more research is needed into the role of disease-associated proteins in non-muscle tissues, the pathological consequences of mutated proteins, and response of these tissues to experimental drug treatments in preclinical models.

To address aspects of these and other outstanding questions that cannot be answered using currently available models, researchers are turning to newly developed strategies to model core myopathies.

## New strategies to study core myopathies

There is no cure for core myopathies, despite tremendous efforts in preclinical and clinical testing. To increase the translation of novel therapeutics, new models that synergize with currently available ones to better recapitulate the spectrum of patient-specific pathologies and enable drug screening are greatly needed. One area of effort is focused on the development of more clinically relevant *in vitro* models that quantifiably recapitulate disease phenotype. In the study of core myopathies, these models are especially necessary to characterize disease progression in human cells. Here, we describe some promising strategies for studying core myopathies in human cells.

### Immortalized myoblast cell lines

As discussed above, transgenic mice and their cell derivatives are a good model to investigate the pathophysiology of muscle diseases. However, the murine phenotype may differ from human probands, as described in the ‘Animal models of core myopathies’ section. Moreover, it is necessary to consider the inter-species differences that can affect experimental findings. For this reason, patient-derived primary cells are a valuable tool to model diseases, although their use is limited both by the difficulty and ethical considerations of obtaining the sample from the patient, and by the short lifespan of primary cell lines *in vitro*, which can be even shorter for pathological cells (Blau et al., 1983; Renna et al., 2014).

Using immortalized patient-derived myoblasts can overcome this. There are multiple approaches to immortalize primary mammalian myoblasts. The first strategy was the transduction of cells with the T antigen from SV40 (Box 3) (Jha et al., 1998), and, more recently, transduction with hTERT (Box 3). The second approach is preferred since it maintains the cell karyotype (Dolivo et al., 2016; Zhu et al., 2007).

Mouly and colleagues co-transduced human myoblasts with hTERT and CDK4 (Box 3), producing a library of more than 35 immortalized human myoblast lines that maintain myogenic potential both *in vivo* and *in vitro* (Mamchaoui et al., 2011). The lines were derived both from healthy donors and patients with 14 different muscle diseases, including Duchenne, facioscapulohumeral, and congenital muscular dystrophies (Mamchaoui et al., 2011). They also developed a CCD line carrying the p.Y4864H mutation that was used by Cacheux et al. (2015) to assess the effect of this patient mutation in 2D myotubes. The authors discovered that p.Y4864H altered RYR1-channel properties and reduced protein expression, as observed in the patient. However, to investigate the effect of the mutation on muscle strength, the investigators moved to a mouse model. This highlights one of the limits of conventional 2D culture – the inability to quantify contractile force, which can be overcome by using 3D *in vitro* systems, as described below.

### Pluripotent stem cell (PSC)-derived myogenic cells

An alternative strategy to overcome the invasiveness of tissue collection and the limited expansion potential of patient-biopsy-derived myoblasts is the use of PSC-derived myogenic cells to model neuromuscular disorders (Chal et al., 2015; Maffioletti et al., 2018; and reviewed extensively in Ortiz-Vitali and Darabi, 2019). In addition to PSC lines derived from skin-biopsy fibroblasts, more recent protocols efficiently generate PSCs from somatic cells collected via non-invasive means, including blood (Chou et al., 2011), nasal brushing/swab (Ono et al., 2012), urine (Zhou et al., 2011) and plucked hair follicles (Novak et al., 2010).

Protocols for deriving myogenic cells from PSCs can be transgene based, whereby myogenic determination genes are overexpressed (Abujarour et al., 2014; Darabi et al., 2012; Maffioletti et al., 2015; Skoglund et al., 2014), or be transgene-free small-molecule-based protocols that mimic embryonic myogenesis (Caron et al., 2016; Chal et al., 2015; Hosoyama et al., 2014). Each approach has advantages: transgene-based protocols have highly synchronized myogenicity, but the proliferation rate and phenotype can be affected by random integration of viral transgenes. Conversely, transgene-free protocols mimic embryonic development, but often require myogenic population purification (Kim et al., 2017), and the resulting myofibers appear more fetal-like (Caron et al., 2016; Chal et al., 2015). Nevertheless, PSC-derived myogenic cells have been used successfully to model several myopathies, including Duchenne (Chal et al., 2015; Choi et al., 2016; Maffioletti et al., 2018), limb-girdle (Maffioletti et al., 2018) and facioscapulohumeral (Caron et al., 2016) muscular dystrophies, myotonic dystrophy (Mondragon-Gonzalez and Perlingeiro, 2018), laminopathies (Steele-Stallard et al., 2018), and motor neuron diseases (Osaki et al., 2018).

The production of recapitulative PSC-derived muscle models depends, in part, on the generation of mature myofibers that resemble the native tissue architecture (Smith et al., 2016). As noted above, a critical aspect for modeling core myopathies is the formation of mature SR and T-tubules with RYR1-DHPR clustering. Despite the presence of myofibrils and spontaneous myotube contraction (Chal et al., 2015; Maffioletti et al., 2015), ultrastructural analysis has revealed inconsistent sarcomere organization unless cultured for extended periods (Jiwlawat et al., 2017). Two recent studies from Tabti and colleagues have assessed the development of ECC components in PSC-derived myotube cultures (Lainé et al., 2018; Skoglund et al., 2014). They show that both embryonic- and induced-PSC-derived myotubes develop mature myofibrils with aligned sarcomeres; RYR1 and DHPR appear from day 4 (Lainé et al., 2018) and RYR1-DHPR clusters after 2-4 weeks in culture (Skoglund et al., 2014). Myotubes are electrically excitable. This aligns with observations from other laboratories using alternative transgene-based differentiation protocols (Maffioletti et al., 2018; Rao et al., 2018). However, Tabti and colleagues' electrophysiological and Ca^2+^-transient measurements found that, whilst myotubes were functional, action potential kinetics and Ca^2+^ release varied (Skoglund et al., 2014). This correlates with observations that the final steps of myofiber maturation, namely CRU morphology and localization, association of CRUs with myofibrils, and transverse organization of triads at A-I bands (Box 3), were incomplete (Lainé et al., 2018; Rao et al., 2018; Skoglund et al., 2014).

Taken together, these studies show that ECC does not reach full maturity in these PSC-derived myotube models, with CRU morphology resembling aneural skeletal muscle (Schiaffino and Settembrini, 1970). Moreover, myotubes formed in 2D culture from PSC-derived progenitors have an immature perinatal morphology (Chal et al., 2015), striated patterning is rarely observed (Chal and Pourquié, 2017), and myofibers often have irregular Z-disks, fail to form T-tubule networks and the triad-like structures are limited to the subsarcolemmal space (Skoglund et al., 2014). Researchers must consider these inconsistencies of PSC-derived myotube cultures when developing disease models of core myopathies where abnormal ECC is a major pathological feature.

Achieving full maturation of human PSC-derived myotubes is expected to help fill the many gaps in our knowledge, such as modeling core development, mitochondrial depletion and Ca^2+^ handling, in real-time. Furthermore, PSC and immortalized myoblast lines can be combined with CRISPR/Cas9 gene editing to characterize mutation-specific phenotypes on the same genetic background (isogenic lines; Table 1) and screen therapeutic strategies in a more relevant manner, as for muscular dystrophies (Li et al., 2015; Turan et al., 2016; reviewed in Ortiz-Vitali and Darabi, 2019). In the next section we discuss advances in 3D human skeletal muscle methodologies that are expected to improve myofiber maturation and allow for functional assessment in culture.

### 3D human cell culture models

**Fig. 4.**
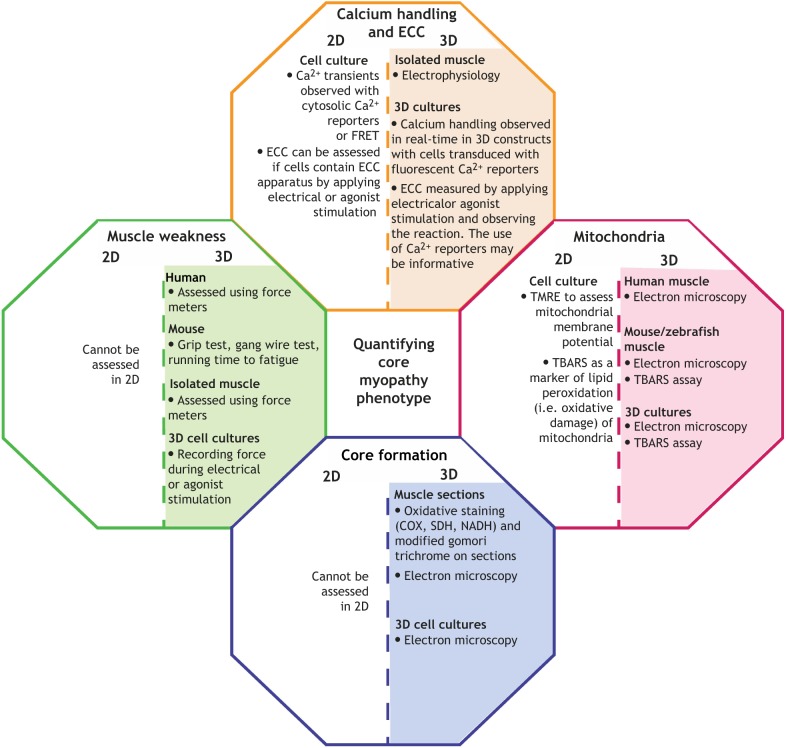
**The different methods in which to quantify the pathology of core myopathies.** COX, cytochrome oxidase; ECC, excitation-contraction coupling; FRET, fluorescence resonance energy transfer; NADH, nicotinamide adenine dinucleotide; SDH, succinate dehydrogenase; TBARS, thiobarbituric acid-reactive substances; TMRE, tetramethylrhodamine ethyl ester.

Animal models have provided a wealth of core myopathy knowledge, but their use is limited by ethical and economic considerations (Pampaloni et al., 2007). By contrast, 2D human cell culture systems are cheap and easy to work with, and minimize ethical concerns, but lack tissue-specific architecture and the mechanical and biochemical signaling that characterize adult human skeletal muscle (Pampaloni et al., 2007). Thus, our own laboratories and others are implementing 3D human cell culture methodologies to boost myofiber structural maturation *in vitro*, a critical requirement in the study of ECC (Bachmann et al., 2019) (Fig. 4).

Generally, 3D skeletal muscle cell culture involves the encapsulation of muscle progenitor cells within a biomaterial and then deposition of these into a culture well containing two attachment points that mimic the tendons to establish uniaxial tension and drive the self-organization of aligned, multinucleated myofibers in the 3D tissue construct. Unlike 2D culture, in which cells bind a substrate only on one side and maturation is affected by aberrant signaling from the cell surface to the nucleus (Baker and Chen, 2012), a 3D matrix allows the cells to spatially organize and assemble into architectures closer to the native physiological conditions (Smith et al., 2016).

Myofilament directionality is fundamental for skeletal-muscle functioning. This anisotropy is achievable in monolayers using patterning methodologies to control cell culture topography (Sengupta et al., 2012) and can be induced in 3D constructs, as described above, by providing anchoring points to establish uniaxial tension as the cells remodel the biomaterial surrounding them. The most commonly used anchor structures for human 3D skeletal muscle cultures are flexible posts or pillars (Eschenhagen and Zimmermann, 2005; Afshar et al., 2019 preprint; Legant et al., 2009; Vandenburgh et al., 2008), but simpler approaches, such as affixing pieces of hook-and-loop fastener (such as Velcro™) to each side of a culture dish, achieve a similar goal (Bell et al., 1979; Vandenburgh et al., 1988). Myotubes that form in engineered 3D human skeletal-muscle tissue systems are structurally more mature than those in conventional 2D culture, and do not need specialized culture supplements (Falcone et al., 2014; Guo et al., 2014; Mills et al., 2019; Pimentel et al., 2017). Specifically, 3D-cultured myofibers express higher levels of adult forms of myosin heavy chains (Afshar Bakooshli et al., 2019), they express SERCA2 and functional RYRs (Madden et al., 2015; Shima et al., 2018), can undergo repeated rounds of contraction in response to electrical and chemical stimulation, and exhibit *in vivo*-like Ca^2+^ handling (Madden et al., 2015).

Abnormal Ca^2+^ transients and muscle weakness are key features of core myopathies (Jungbluth et al., 2011) that can be assessed in 3D-cultured human myofibers. Whilst it is possible to observe Ca^2+^ handling in monolayers (see ‘*In vitro* models of core myopathies’ section), measurements generally cannot be repeated over time. By contrast, transducing 3D myoblasts with a Ca^2+^ reporter, such as GCaMP6 (Box 3) (Chen et al., 2013), allows non-invasive and real-time monitoring of Ca^2+^ transients. This technique was used by Madden and colleagues to assess Ca^2+^ handling in 3D myofibers following caffeine-induced RYR1 activation (Madden et al., 2015). Another advantage of 3D models is the possibility to evaluate force across the anchoring posts. Quantification of post deflection directly measures contractile force after chemical or mechanical stimulations (Afshar et al., 2019 preprint; Legant et al., 2009; Vandenburgh et al., 2008).

Despite the advantages, there is still no evidence that 3D culture systems are the ideal platform to explore aspects of CCD. Interestingly, amorphous central cores that lack enzymatic activity, the defining feature of core myopathies, are most often observed in type 1 (slow twitch) myofibers (Sato et al., 2008; Wu et al., 2006). The premature loss of the most mature myotubes in conventional 2D culture impedes the study of mature fiber types, and this can partially be overcome by 3D modeling (Shima et al., 2018). The development of a human model in which different fiber types develop would give insight into why these myofibers are more prone to core lesions. However, it is also quite possible that 3D human myofiber culture systems require additional modifications to allow cores, and other features of CCD, to form in a dish. Although 3D cultures are enriched for larger-diameter myofibers than those observed in conventional 2D cultures at similar time points (Afshar Bakooshli et al., 2019; Shima et al., 2018), *in vitro* myofibers are still smaller than adult human skeletal-muscle ones. Thus, several strategies are being pursued to boost 3D-cultured human myotube maturation. For example, electrical and mechanical stimulation can induce tissue maturation, increasing myosin heavy chain expression and myoblast differentiation *in vitro* (Niu et al., 2013; Player et al., 2014), as well as other tissue maturation benefits, i.e. improved metabolic flux, increased Ca^2+^ transient amplitude, tetanic force and fatigue resistance (Davis et al., 2019; Khodabukus et al., 2019; Martin et al., 2017; Mills et al., 2019; Takahashi et al., 2018). These simulated ‘exercise protocols’ have greatly increased myotube width and force (Powell et al., 2002; Raman et al., 2016). Another approach to promote 3D muscle maturation is co-culture with supporting cells such as motor neurons (Afshar Bakooshli et al., 2019; Happe et al., 2017). Indeed, an increasing number of studies are focusing on uncovering the influence of other tissue-resident cell populations on human skeletal-muscle maturation and function (Bersini et al., 2018; Juhas et al., 2018; Kalman et al., 2015; Maffioletti et al., 2018; Osaki et al., 2018). Nevertheless, the presence of multiple cell types makes it harder to find the optimal culture conditions (Esch et al., 2014), and researchers must carefully evaluate the complexity required to answer their questions, or consider whether one can replace the activity of the added cell type with the addition of a purified protein or pharmacological inhibitor to the culture medium.

A further advantage of 3D cell culture models is the possibility to decrease the intrinsic variability often associated with animal models; replicate number is limited to the expansion potential of the cell type used, and variability can be further decreased through the implementation of robotics and their integration with miniaturization strategies (Afshar et al., 2019 preprint; Capel et al., 2019; Laternser et al., 2018; Mills et al., 2019; Osaki et al., 2018; Vandenburgh et al., 2008). Computer-aided designs make it possible to build up cell-laden hydrogels layer by layer, in which culture thickness can be controlled by modulating the number of printed layers (Haycock, 2011; Kim et al., 2018). Platform miniaturization can be aided by bio-printing (Box 3) to reduce construct size without losing precision. Bio-printed microtissues can be integrated with pillars or posts for simulated ‘exercise protocols’ and force assays (Kim et al., 2018). Alternatively, one can envision the use of robotics to perform most culture and information-capture steps in multi-well platforms. In this way, 3D human skeletal-muscle microtissue technologies function as a logical intermediary to bridge 2D screens and animal toxicology studies.

Therefore, 3D *in vitro* models are a valuable tool to discover physiological and pathological mechanisms in skeletal muscle. They may be successfully utilized to study core myopathies, providing an amenable platform to evaluate Ca^2+^ fluxes, muscle strength and better fiber maturation compared to traditional 2D culture (Fig. 4).

## Concluding remarks

In the past decades, research into core myopathies has provided great insight into the cause and progression of this subgroup of congenital myopathies. However, major gaps in our understanding of pathogenic mechanisms remain, due in part to a lack of recapitulative models, especially for non-*RYR1*-associated core myopathies. Dowling and colleagues recently noted that the time for research into congenital myopathies ‘is now’ (Dowling et al., 2018). When generating 3D *in vitro* or *in vivo* models, researchers should take care to holistically model the various aspects of mutation-specific disease mechanisms and progress with those models that best recapitulate the tissues and structures affected in the human disorder. In turn, these models will allow the search for effective treatment in these disorders and pave the way to the ‘therapeutic era’ of core myopathies, allowing treatment before clinical signs or muscle damage become apparent.
